# Internet addiction and its association with quality of life in patients with major depressive disorder: a network perspective

**DOI:** 10.1038/s41398-022-01893-2

**Published:** 2022-04-04

**Authors:** Wei Bai, Hong Cai, Siqi Wu, Ling Zhang, Ke-Xin Feng, Yu-Chen Li, Huan-Zhong Liu, Xiangdong Du, Zhen-Tao Zeng, Chang-Mou Lu, Wen-Fang Mi, Lan Zhang, Yan-Hong Ding, Juan-Juan Yang, Todd Jackson, Teris Cheung, Feng-Rong An, Yu-Tao Xiang

**Affiliations:** 1 Unit of Psychiatry, Department of Public Health and Medicinal Administration, & Institute of Translational Medicine, Faculty of Health Sciences, University of Macau, Macao SAR, China; 2 Centre for Cognitive and Brain Sciences, University of Macau, Macao SAR, China; 3 Institute of Advanced Studies in Humanities and Social Sciences, University of Macau, Macao SAR, China; 4 School of Psychology and Mental Health, North China University of Science and Technology, Tangshan, Hebei Province China; 5 Guangji Hospital Affiliated to Soochow University, Suzhou, Jiangsu Province China; 6 Nanning Fifth People’s Hospital, Nanning, Guangxi Province China; 7 School of Public Health, Lanzhou University, Lanzhou, Gansu Province China; 8 Department of Psychiatry, Xiamen Xianyue Hospital, Xiamen, China; 9 Department of Psychiatry, Chaohu Hospital, Anhui Medical University, Hefei, China; 10 School of Mental Health and Psychological Sciences, Anhui Medical University, Hefei, China; 11 Department of Psychiatry, Lanzhou University Second Hospital, Lanzhou, Gansu Province China; 12 Department of Psychology, University of Macau, Macao SAR, China; 13 School of Nursing, Hong Kong Polytechnic University, Hong Kong SAR, China; 14 The National Clinical Research Center for Mental Disorders & Beijing Key Laboratory of Mental Disorders, Beijing Anding Hospital & the Advanced Innovation Center for Human Brain Protection, Capital Medical University, Beijing, China

**Keywords:** Depression, Addiction

## Abstract

Depressive disorders and internet addiction (IA) are often comorbid. The aims of this study were to examine the network structure of IA in patients with major depressive disorders (MDD) and explore the association between IA and quality of life (QoL) in this population. This was a multicenter, cross-sectional survey. IA and QoL were assessed with the Internet Addiction Test (IAT) and the World Health Organization Quality of Life-brief version, respectively. Node expected influence (EI) was used to identify central symptoms in the network model, while the flow network of QoL was generated to examine its association with IA. A total of 1,657 patients with MDD was included. “Preoccupation with the Internet,” “Job performance or productivity suffer because of the Internet,” and “Neglect chores to spend more time online” were central symptoms. The symptom “Form new relationships with online users” had the strongest direct positive relation with QoL, while “Spend more time online over going out with others” and “Job performance or productivity suffer because of the Internet” had the strongest direct negative relations with QoL. Neglecting work caused by IA correlated with QoL, while making friends online appropriately was related to better QoL among MDD patients. Appropriate interventions targeting the central symptoms may potentially prevent or reduce the risk of IA in MDD patients.

## Introduction

Over the past decade, online activity has become a central aspect of daily life [1]. With the proliferation of internet users, risks of Internet overuse and even internet addiction (IA) have been growing [2]. A systematic review showed that the prevalence rate of IA ranged from 0.5 to 40.0% among adolescents and adults, with a pooled prevalence of 7.02% (95% confidence interval (CI): 6.09–8.08%) [3].

IA refers to online-related compulsive behavior reflecting an inability to control an individual’s use of the internet [4] and is closely associated with psychiatric problems (e.g., depression, anxiety, and harmful alcohol use) [5, 6], particularly depression [7]. Epidemiological studies have found that the prevalence of IA in patients with depression ranges from 36.0 to 58.6% [8, 9]. The relationship between IA and depression is bidirectional. For instance, individuals with excessive internet use often suffer from more severe depressive symptoms [10], while depressive symptoms are associated with an increased risk of problematic internet use (e.g., preference for online social interaction and increased use of the internet for mood regulation) [11]. Moreover, the internet is a major source of leisure activity and a strategy used to cope with emotional and social difficulties, especially among those who experience depression [7]. Excessive internet use without any limits may result in IA, exacerbations of existing depressive symptoms, and a lowered quality of life (QoL) [12]. Additionally, depressive symptoms can significantly affect QoL among patients with major depressive disorder (MDD), who often report a poorer QoL compared to patients with other severe mental illnesses (e.g., schizophrenia and bipolar disorder) [13, 14]. To reduce negative outcomes caused by IA, it is important to understand specific symptoms of IA that have the strongest links with QoL among individuals with depression.

The Internet Addiction Test (IAT) [4] is the most commonly used assessment tool for IA symptoms. IA may be driven by various factors and manifested in behaviors such as escape, compulsion, neglecting duties, anticipation, lack of control, and social avoidance [4]. However, traditionally IA is evaluated using total scores on IA measures and most studies have focused on the general prevalence of IA and its correlates [15, 16]. Unfortunately, the effectiveness of treatments for IA at the syndrome level based on scale total scores may be mitigated because such approaches fail to consider inter-relationships between individual IA symptoms [17].

Network analysis is a novel approach used to explore and illuminate associations of individual psychiatric symptoms. In network analysis theory, a psychiatric disorder/syndrome can be modeled as arising from a network of interacting and mutually reinforcing symptoms [18]. Network analysis can identify central symptoms (i.e., symptoms with the strongest connections with other symptoms) that are most influential and have the strongest impact within a network of symptoms [19], and the association between two symptoms can be calculated after controlling for other symptoms. Moreover, unlike traditional statistical methods (e.g., factor analysis and conventional regression analysis), dynamic and reciprocal relationships between symptoms can be identified in network analysis in line with clinical findings [20]. Central symptoms are potentially useful for understanding mechanisms involved in the onset and maintenance of a disorder/syndrome and serve as promising intervention targets that result in more efficient treatment outcomes [21].

Previous studies have examined symptoms of depression, anxiety, and autism spectrum disorder (ASD) using network analysis [17, 22]. However, few network analysis studies have focused on comorbid IA symptoms in patients with psychiatric disorders. One network analysis revealed that defensive, secretive behaviors and concealment of internet use were central symptoms among 108 adolescents with ASD [17]. To date, however, no studies have explored the network structure of IA symptoms or its links with measures of functioning (e.g., QoL) among patients with MDD.

To address this gap, we conducted network analysis to examine comorbid IA symptoms in a large sample of stable patients with MDD, and also explored relations between IA symptoms and QoL.

## Methods

### Participants

This multicenter cross-sectional study was conducted between September 21, 2020 and August 23, 2021 in six tertiary psychiatric hospitals and psychiatric units of general hospitals in China that are located in the north, east, south, and west of China, enhancing the geographic representativeness of the study sample. To avoid transmission of COVID-19, an online survey using the WeChat-based QuestionnaireStar application was adopted following previous studies [23, 24], instead of traditional face-to-face interviews. Patients needed to report their health status during the COVID-19 pandemic when they entered participating hospitals using WeChat; therefore all patients were presumed to be WeChat users. Both inpatients and outpatients were consecutively recruited with the following inclusion criteria: (1) aged 18 years or above, (2) a primary diagnosis of MDD according to the International Statistical Classification of Diseases and Related Health Problems, 10th Revision (ICD-10) [25], (3) clinical stability judged by treating psychiatrists (i.e., the dose change of their antidepressant medications was less than 50% during the past three months) [26, 27], and (4) ability to understand the survey purpose and complete the assessment. All participants and/or their legal guardians provided electronic written informed consent. The study protocol was centrally approved by the medical ethics committees of Beijing Anding hospital and respective hospitals.

### Data collection and measurements

The data collection form was designed, and then a QR code was generated. Participants who met the inclusion criteria scanned the QR code using a personal or guardian smartphone to complete the assessment when they attended on a voluntary and confidential basis after providing the electronic written informed consent.

Basic sociodemographic data including age, gender, marital status, and education level, were collected. IA symptoms were assessed using the Internet Addiction Test (IAT) questionnaire [4, 28], a 20-item self-report scale measuring characteristics and behaviors (i.e., compulsivity, escapism, and dependency) related to IA during the past month. The IAT consists of six domains, including Excessive use (items 1, 2, 14, 18, and 20), Salience (items 10, 12, 13, 15, and 19), Neglect work (items 6, 8, and 9), Anticipation (items 7 and 11), Lack of control (items 5, 16, and 17), and Neglect social life (items 3 and 4). Items are rated on a 5-point Likert scale and can generate a maximum score of 100, with higher scores indicating a higher overall level of IA severity [4]. Global QoL was assessed from total scores of the first two items of the World Health Organization Quality of Life-BREF (WHOQoL-BREF) Chinese version, with a higher score representing a higher QoL [29]. Chinese versions of the IAT questionnaire [30] and WHOQoL-BREF [31] have been validated in Chinese populations.

### Statistical analysis

All statistical analyses were conducted using R program, v4.1.1 [32]. In network parlance, each node represents an individual item from a measure and the edge between two nodes indicates the partial association between them. Green (red) edges illustrate positive (negative) associations, and thicker, more saturated edges reflect stronger associations [33].

The network of IA symptoms was estimated and visualized by the packages *bootnet* v1.4.3 [34] and *qgraph* v1.6.9 [33]. As recommended previously [35], relations between IA symptoms were examined with polychoric correlations when taking Likert scale-type variables into account. To estimate and ensure a sparse and interpretable network model, the graphical least absolute shrinkage and selection operator (LASSO) statistical regularization technique was applied in tandem with the Extended Bayesian Information Criterion (EBIC) model selection [33]. A tuning hyperparameter was set to 0.05, which has been widely used in estimating network structures [36, 37].

To quantify which node displays the highest connectivity in the network, the centrality index, expected influence (EI), was computed [38]. The EI index takes negative associations into account rather than summing up the absolute edge weight, which is recommended in a network with negative edges [38]. Predictability was calculated using the package *mgm* v1.2–12 [39] and indicated the extent to which a node was predicted by all its neighboring nodes. Predictability was expressed as a pie chart on the border of each node. The associations of mean IAT item scores with EI and predictability were examined using Spearman rank-order correlations. Direct and indirect influences of IA symptoms on QoL were plotted using the function *flow* in the package *qgraph* [33].

To evaluate the robustness of the estimated network, centrality stability was examined using the correlation stability coefficient (CS-coefficient). A CS-coefficient value above 0.25 indicates that observed network model results are stable, though traditionally, CS-coefficient values above 0.5 are preferable. A bootstrapped difference test was conducted to assess the robustness of node EIs and edges. Differences were significant between two nodes or two edges if zero was not included in the 1000-bootstrap 95% confidence interval (CI). Edge accuracy was estimated with bootstrapped 95% CIs; a narrower CI suggests a more reliable network. These procedures were conducted using the package *bootnet* v1.4.3 [34]. Finally, following previous studies [40, 41], the network model was re-estimated after controlling for basic socio-demographic variables (e.g., age, gender, marital status, and education level) using the package *mgm* v1.2–12 [39], and then compared with the original network with respect to EIs using Spearman rank-order correlation coefficients.

## Results

### Study sample and descriptive statistics

From 1698 participants who were initially invited, the final sample comprised 1657 MDD patients who fulfilled all study entry criteria and completed the assessment, for a participation rate of 97.6%. The mean sample age was 31.58 years (standard deviation (SD) = 14.54 years), 71.6% were women, 39.8% were married, and more than half (51.4%) had an education level of a college degree or above. The prevalence of IA (total score of ≥50) was 28.1% (95% CI: 26.0–30.3%). The mean score of each item in the IAT questionnaire is shown in Table 1.

**Table 1:** Item statistics.

Items	Abbreviations	Mean (SD)	EI^a^	Predictability
Stay online longer than you intend	IAT1	2.70 (1.27)	0.74	0.44
Neglect chores to spend more time online	IAT2	2.16 (1.16)	1.07	0.57
Prefer the excitement online to the time with others	IAT3	2.33 (1.29)	0.93	0.54
Form new relationships with online users	IAT4	1.71 (0.96)	0.43	0.16
Others complain about your time spent online	IAT5	2.14 (1.25)	1.02	0.51
School grades suffer due to internet use	IAT6	1.92 (1.14)	1.05	0.58
Check email/SNS before doing things you need to do	IAT7	1.79 (1.06)	0.70	0.34
Job performance or productivity suffer because of the Internet	IAT8	1.74 (1.03)	1.09	0.56
Become defensive/secretive about the internet use	IAT9	2.37 (1.30)	0.61	0.31
Soothe disturbing thoughts using the Internet	IAT10	2.41 (1.31)	0.98	0.54
Anticipation for future online activities	IAT11	2.02 (1.19)	1.07	0.56
Life boring and empty without the Internet	IAT12	2.41 (1.31)	0.97	0.55
Snap or act annoyed if bothered while being online	IAT13	1.82 (1.06)	1.07	0.58
Sleep loss due to late-night logins	IAT14	2.18 (1.21)	0.96	0.55
Preoccupation with the Internet	IAT15	2.04 (1.09)	1.11	0.59
Request an extension for longer time spent online	IAT16	2.20 (1.18)	1.00	0.58
Failure to cut down the time spent online	IAT17	1.87 (1.11)	0.98	0.57
Conceal the amount of time spent online	IAT18	1.62 (1.00)	0.85	0.47
Spend more time online over going out with others	IAT19	1.99 (1.23)	1.00	0.57
Depressed/moody/nervous only while being offline	IAT20	1.68 (1.04)	1.01	0.55

*EI* expected influence, *IAT* Internet Addiction Test, *SD* standard deviation.

^a^The value of EI is shown as raw data.

### Network structure and centrality

**Fig. 1:**
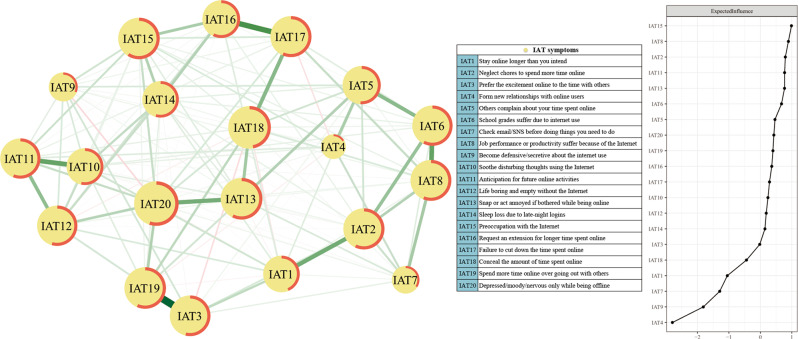
Network structure of internet addiction symptoms in patients with major depressive disorder. IAT internet addiction test.

Figure 1 depicts the network structure of IA symptoms in MDD patients. A total of 190 edges (20 × (20 − 1)/2) were estimated; of these, 135 edges had non-zero weights. The connection between symptoms, “Prefer the excitement online to the time with others” (IAT3) and “Spend more time online over going out with others” (IAT19) was the strongest edge, followed by connections between “Request an extension for longer time spent online” (IAT16) and “Failure to cut down the time spent online” (IAT17), between “School grades suffer due to internet use” (IAT6) and “Job performance or productivity suffer because of the Internet” (IAT8), and between “Soothe disturbing thoughts using the Internet” (IAT10) and “Anticipation for future online activities” (IAT11).

On the right panel of Fig. 1, the EI of 20 symptoms in the IAT network is plotted by order of respective value. The symptom, “Preoccupation with the Internet” (IAT15) had the highest EI value and was the most central symptom in the network model, followed by symptoms, “Job performance or productivity suffer because of the Internet” (IAT8), and “Neglect chores to spend more time online” (IAT2). The raw data of EI values are shown in Table 1. In terms of the predictability index, an average of 51% of the variance could be potentially explained by neighboring nodes (*M*_predictability_ = 0.51 ± 0.11). The item, “Preoccupation with the Internet” (IAT15), had the highest predictability value (59%) in the network (Table 1). There were no significant associations of item mean level with EI and predictability (EI and item mean level: *r*_s_ = −0.158, *p* = 0.506; predictability and item mean level: *r*_s_ = −0.061, *p* = 0.799).

**Fig. 2:**
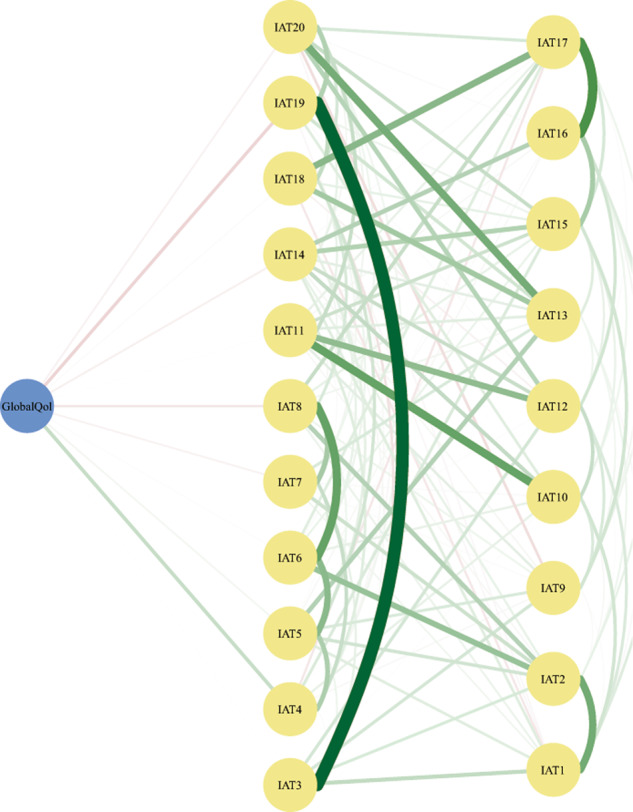
The flow diagram of network showing how the quality of life is connected to internet addiction symptoms. Note: items in the middle of the figure indicate the direct connection to quality of life and items located on the right represent the indirect connection.

As shown in Fig. 2, the flow network of connections between QoL and IA highlights individual IA symptoms directly and indirectly related to QoL. Of the IA symptoms directly related to QoL, “Form new relationships with online users” (IAT4) had the strongest positive association while “Spend more time online over going out with others” (IAT19) and “Job performance or productivity suffer because of the Internet” (IAT8), had the strongest negative associations with QoL.

### Network stability and accuracy

**Fig. 3:**
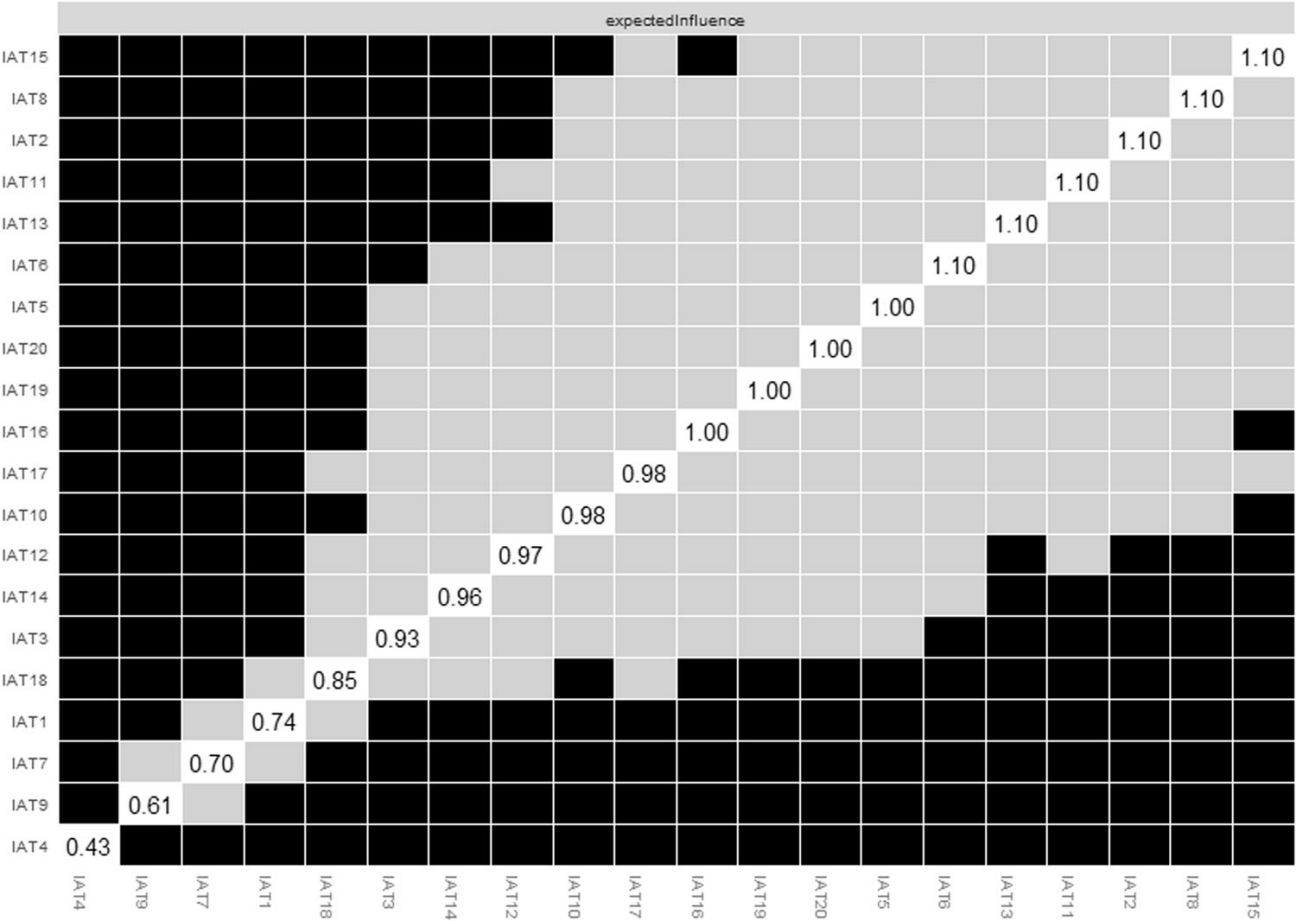
Estimation of node expected influence difference by bootstrapped difference test. Note: Gray boxes indicate nodes that do not significantly differ from one-another. Black boxes represent nodes that differ significantly from one another (α = 0.05). White boxes show the values of node strength.

As presented in Fig. S1, the CS-coefficient of EI calculated by the case dropping bootstrap method was 0.75, indicating that the network remained stable after dropping 75% of the sample. Bootstrapped difference tests for node EIs showed central symptoms were significantly different from most nodes, indicating the primary results are robust (Fig. 3). In terms of accuracy of the observed network model, bootstrapped 95% CIs of edges were narrow, and bootstrapped difference tests for edge weights showed that most comparisons were significantly different; in particular, the strongest edges were significantly different from most edges (Figs. S2 and S3), a pattern that also indicates primary results are trustworthy.

### The effect of covariates

Previous studies [40, 41] found that age, gender, marital status, and/or an educational level were significantly associated with IA [42, 43]. Hence, the network model was re-estimated after controlling for age, gender, marital status, and educational level. Compared with the original network, no significant change was found (*r*_s_ = 0.87, 95% CI: 0.55–0.85) (Fig. S4), underscoring how the observed network model was not significantly influenced by demographics.

## Discussion

This was the first study to explore the network structure of IA symptoms in MDD patients and its association with QoL in this population. Within the entire IA symptom network, the association between symptoms “Prefer the excitement online to the time with others” (IAT3) and “Spend more time online over going out with others” (IAT19) had the strongest edge, in contrast to previous findings in adolescents with ASD [17] wherein “Conceal the amount of time spent online” and “Spend more time online over going out with others” had the strongest connection. This discrepancy may be due to the different features of the two study samples. For example, adolescents may be more likely than adults to conceal their internet use to avoid monitoring of and unnecessary conflicts with their parents who often limit their internet use [17]. Conversely, MDD patients may be less likely to seek help from others due to specific symptoms of depression (e.g., loss of energy and motivation) as well as stigma and discrimination associated with having a psychiatric disorder [44]. Hence, depressed persons may turn to social media for health information and communications with friends [45], resulting in the strong association between “Prefer the excitement online to the time with others” (IAT3) and “Spend more time online over going out with others” (IAT19).

We also found that “Preoccupation with the Internet” (IAT15) was the most central symptom with the highest EI value and predictability in MDD patients. This symptom is a facet of the IAT dimension, “Cognitive salience,” which has been defined as how “one feels preoccupied with the internet when off-line or fantasizes about being online” [46]. This finding is consistent with proposed diagnostic criteria for IAD and a major feature for this disorder [47]: “preoccupation/salience.” In MDD patients, cognitive dysfunction is a core disturbance [48] that can persist both during acute depressive episodes and remission [49, 50]. A meta-analysis [51] revealed that problematic internet use is associated with cognitive impairments (e.g. deficits in inhibitory control and decision making), and could result in loss of control in internet use. Together, these lines of research may help to explain the emergence of “Preoccupation with the Internet” (IAT15) as the most central feature of IA in MDD patients. To alleviate this symptom, empirically-supported psychosocial interventions such as cognitive-behavioral therapy (CBT) should be adopted [52]. CBT may aid in helping MDD patients with elevations in IA to learn how to control dysfunctional thoughts and feelings that impair functioning and trigger the impulse to cope by escaping from the real world to the virtual world [53].

The node “Job performance or productivity suffer because of the Internet” (IAT8) was another central symptom in the model, reflecting the negative effects of IA on daily work. This finding converges with previous findings linking excessive smartphone use (a proxy to Internet use) to reduced work productivity in employees [54]. There are several reasons for the negative influence of IA on job performance. First, IA could interfere with healthy sleep patterns [55]. Employees with IA may spend more time on the Internet at night, causing irregular or insufficient sleep and daytime fatigue that hamper job performance due to impaired concentration, drowsiness, and insufficient energy during working hours. Second, people with IA direct excessive attention towards online pursuits at the cost of investing sufficient attention and interest in job-related responsibilities and teamwork [56]. Moreover, previous studies found that MDD-induced cognitive dysfunction has a direct negative influence on job performance and productivity [57, 58], which could worsen due to comorbid IA [59]. Therefore, MDD patients may tend to present with “Job performance or productivity suffer because of the Internet” as a central symptom of IA. Similarly, adverse effects of IA on job duties and domestic responsibilities [60] were confirmed in this study with the emergence of “Neglect chores to spend more time online” (IAT2) as another central symptom in the current network model.

The flow network of QoL and IA symptoms underscored both positive and negative associations between internet use and QoL among patients with MDD. The item, “Form new relationships with online users” (IAT4) had the highest positive association with QoL, indicating that making friends online corresponds with improved QoL in MDD patients. One longitudinal study found that internet use could reduce the severity of depression, in part, due to improved social relationships [61]. Another longitudinal study found that internet use for communication with friends/family had a protective role in the development of clinical depression [62]. Because reductions in energy and activity, including social activity, are prominent features of MDD (American Psychiatric Association, 2013) and stigma or discrimination associated with having a psychiatric disorder can impede in-person social contact [63], the capacity to make new friends online may increase opportunities to receive social support and companionship from others among patients with MDD.

However, when use of the internet to make new online friends is excessive, there is an increased risk for over-indulgence in the virtual world to the neglect of sustaining bonds with families and friends in the real world and responsibilities related to work and non-work contexts [54, 56]. Indeed, excessive use of the internet to make friends and/or for other reasons may help to explain why the symptoms, “Spend more time online over going out with others” (IAT19) and “Job performance or productivity suffer because of the Internet” (IAT8) were negatively associated with QoL in this study.

The average predictability of 51% in this study indicated that just over half of the variance in IA symptoms could be accounted for by the observed network model while the remaining variance is attributable to other unmeasured IA-related factors such as comorbid psychiatric syndromes or symptoms [64, 65] and measurement error. Moreover, as recommended previously [40], we examined the association between expected influence and predictability with the mean IAT item score but found no significant associations. These findings underscored an advantage of network analysis wherein potential insights into strengths of inter-relations between individual symptoms within the symptom network model can be garnered compared to traditional approaches that rely upon IAT total scores or IAT item mean scores without adjusting for observed variability of differences in relative weights of particular items or edges within a network model. For example, “Preoccupation with the Internet” (IAT15) had the highest predictability and EI in the network model but its impact could have been obscured using the traditional approach because the mean of IAT15 was not among the highest IAT item scores (Table 1).

Strengths of this study included its large sample size, multi-center study design, highly reliable results, and use of network analysis to elucidate key symptoms of IA for people who experience MDD. Several limitations should also be acknowledged. First, due to the non-experimental design, causal associations between IA symptoms and QoL could be not inferred. Second, due to the COVID-19 pandemic, an online self-report assessment approach was used to ensure participant safety. Consequently, certain response biases (e.g., lack of care in answering questions) or selection biases (e.g., those who used smartphones and/or preferred to use computers were more likely to participate in this study), could not be assessed per interview-based evaluations. Third, because this study focused exclusively upon stable MDD patients, generalizations of findings cannot be made to less stable MDD patients, other psychiatric populations, or the general population. Nonetheless, this study provides foundations for extensions to these groups.

In conclusion, this study underscored how “Preoccupation with the Internet” and impaired functioning (“Job performance or productivity suffer because of the Internet,” “Neglect chores to spend more time online”) are central symptoms of IA among patients with MDD. Furthermore, although making friends online appropriately was related to better QoL in this population, those who reported spending more time online than going out with others and job performance or productivity deficits due to excessive internet use tended to report poorer QoL. To reduce negative outcomes associated with IA, appropriate interventions targeting these central symptoms warrant attention in future work devoted to preventing the development of IA and reducing its current severity among MDD patients.

## Supplementary information

Supplementary material
