# Supplementary material for: Internet addiction and its association with quality of life in patients with major depressive disorder: a network perspective
Source: Transl Psychiatry. 2022 Apr 4;12:138. doi: 10.1038/s41398-022-01893-2 (PMC8977829; doi:10.1038/s41398-022-01893-2)
Supplement: Supplementary file 1 — Supplementary material [file 41398_2022_1893_MOESM1_ESM.docx]

**Supplementary materials**

Supplementary Figure 1. The stability of network using the case-drop procedure (CS-coefficient=0.75)

Supplementary Figure 2. Bootstrapped confidence intervals of edge weights

Supplementary Figure 3. Estimation of edge weight difference by bootstrapped difference test

Supplementary Figure 4. The comparison of networks after including covariates


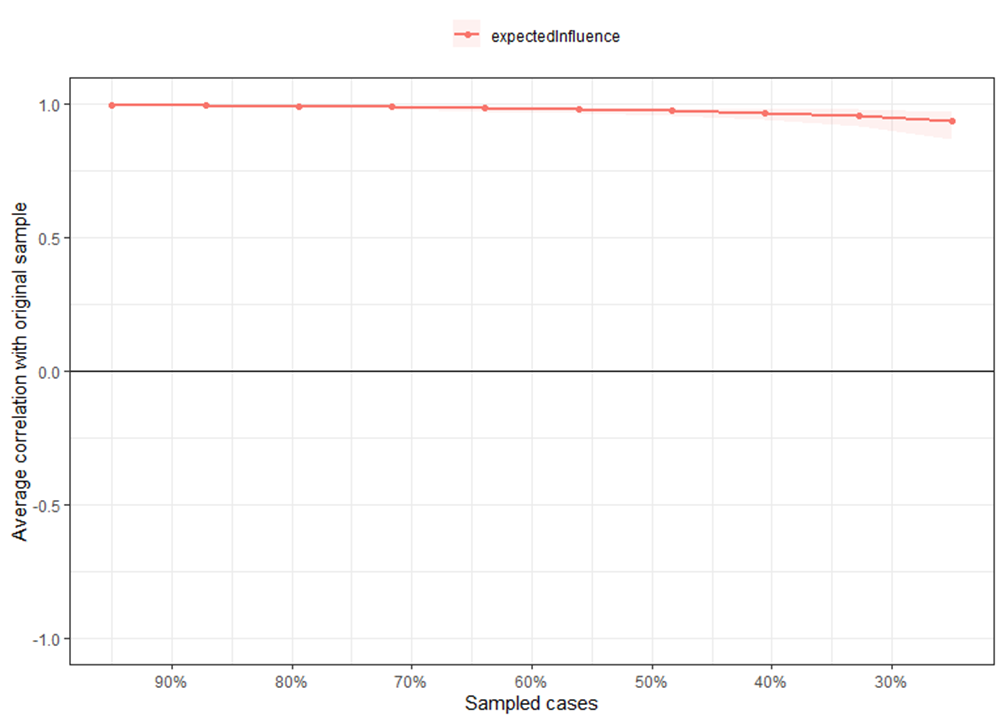


**Supplementary Figure 1. The stability of network using the case-drop procedure. (CS-coefficient=0.75)**

**
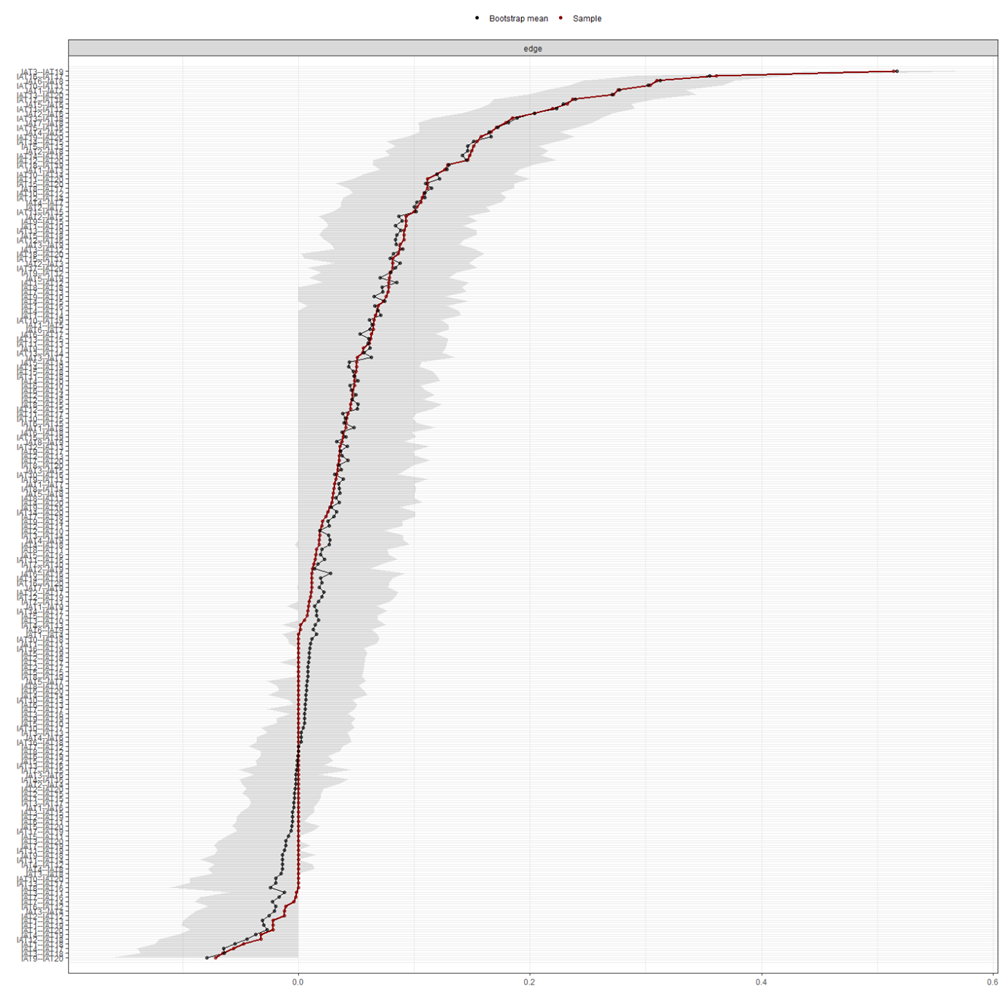
Supplementary Figure 2. Bootstrapped confidence intervals of edge weights**

The black dots indicate the values of each edge weight, ordered from the highest to the lowest value. The gray area represents the 95% Confidence Intervals of edge weights, estimated with the non-parametric bootstrap procedure (Bootnet package). Wide intervals indicate lower stability and narrow intervals indicate higher stability

**
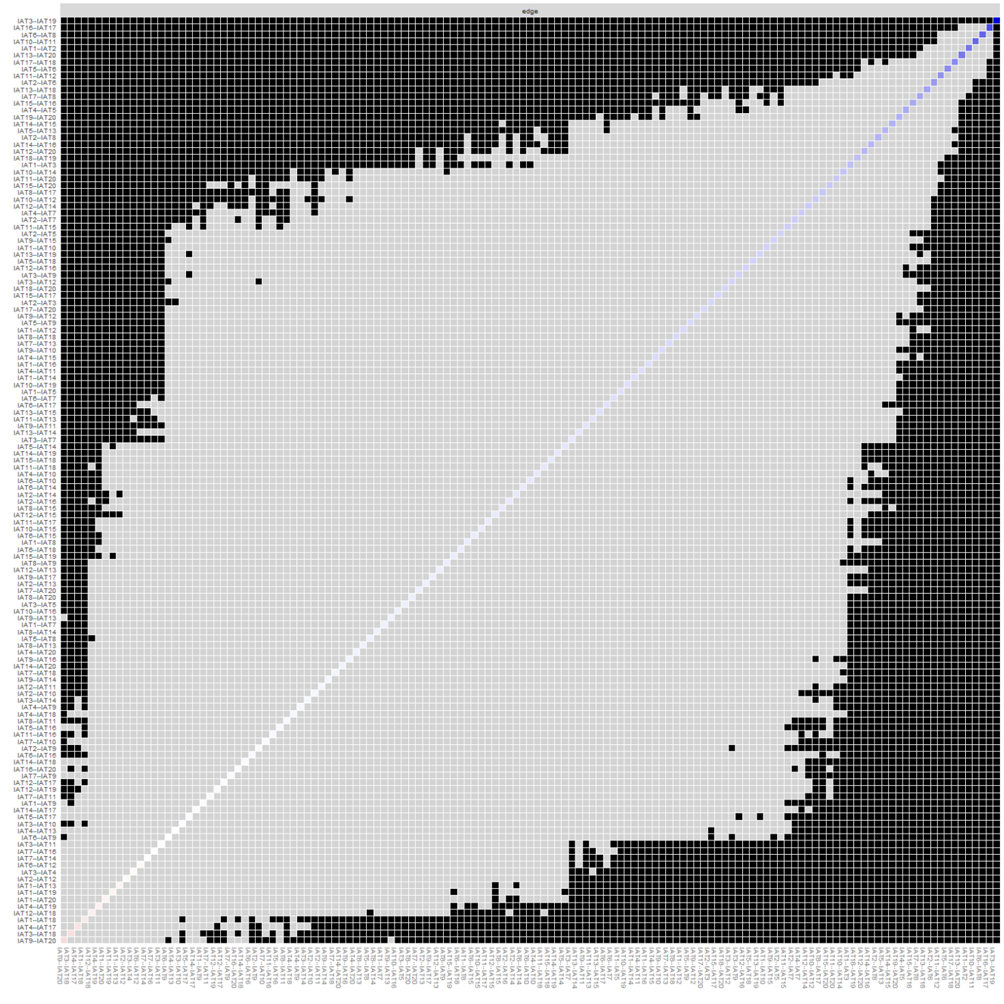
Supplementary Figure 3. Estimation of edge weight difference by bootstrapped difference test**

Bootstrapped difference tests between edge weights in the network. Gray boxes indicate edges that do not significantly differ from one-another. Black boxes represent edges with significant difference from one another (α = 0.05). Blue boxes in the edge-weight plot indicate positive correlations.

**
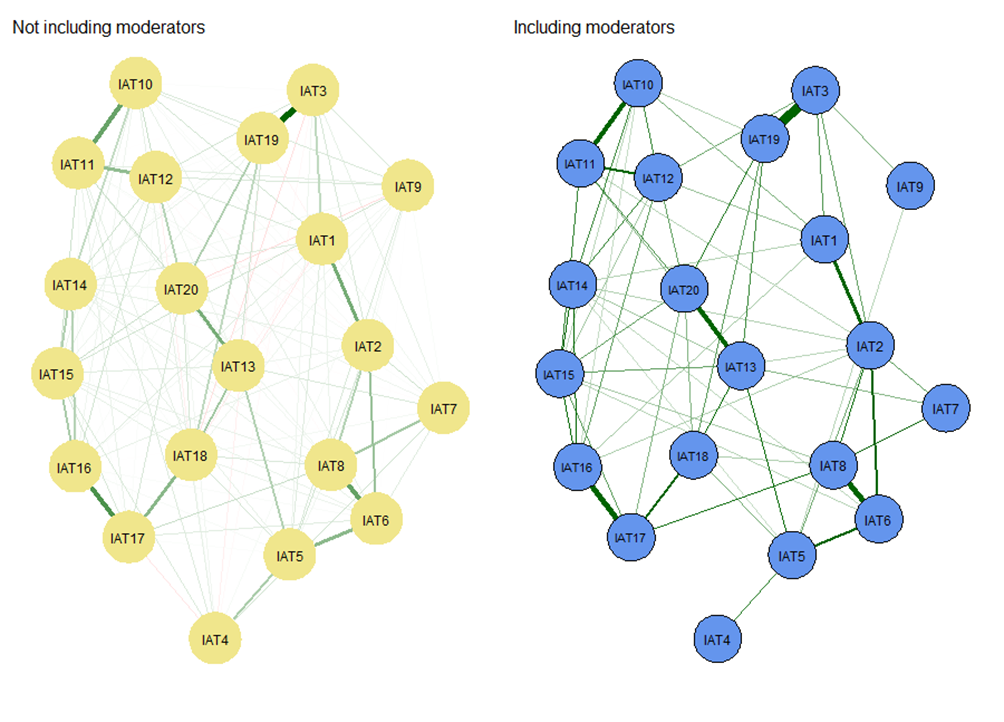
**

**Supplementary Figure 4. The comparison of networks after including covariates**

Note: Variables, including age, gender, marital status and education level, were set as moderators in the right panel of the network.
